# Bilateral septic arthritis of the knee caused by group B streptococci: a case report

**Published:** 2019-04

**Authors:** Seyed Ali Dehghan Manshadi, Ramazanali Alizadeh, Mohammadreza Salehi, Arash Seifi, Mojgan Seifi

**Affiliations:** 1 Department of Infectious Diseases, School of Medicine, Tehran University of Medical Sciences, Tehran, Iran; 2 Department of Orthopedics, School of Medicine, Ahvaz Jundishapur University of Medical Sciences, Ahvaz, Iran

**Keywords:** Septic arthritis, *Streptococcus agalactiae*, Diabetes mellitus

## Abstract

Septic arthritis (SA) remains to be a critical diagnosis for a swollen knee at the emergency department. Here, we report a rare case of bilateral knee arthritis in a 59-year-old diabetic woman who had been immobilized 5 months prior to admission. Her right knee swelling exacerbated in 10 days leading to left knee involvement. In 5 days the clear synovial tap in the first hospital turned purulent in the second hospital and empirical antibiotics get started with high WBC count, dominant neutrophils, and Gram-positive cocci in smear. Knee arthrotomy was performed after 6 days in the third hospital with the same smear results but negative blood and synovial cultures of both knees. When followed in retrograde, two positive blood cultures were reported for *Streptococcus agalactiae* in the second hospital. Vancomycin was changed to ampicillin and symptoms were resolved in 4 weeks. Despite improvement, mobility was not retained. Uncommon etiologic agents of knee arthritis should be in mind specifically in debilitated patients. Timely initiation of proper antibiotics hinders permanent sequels, hence clinicians should be suspicious of such organisms.

## INTRODUCTION

Septic arthritis (SA) is among the major diagnoses of a swollen painful joint at the emergency department. The annual incidence has been reported to be around 2 to 20 per 100,000, while 70 per 100,000 person-year in patients affected with rheumatoid arthritis. Other risk factors include diabetes mellitus, intravenous drug abuse, previous surgery, and alcohol use (1).

*Staphylococcus aureus* remains the top etiologic cause of SA (2). The attributed morbidity and mortality is substantial; treatment is of critical benefit to avert consequences (3). Culture-based diagnosis is not helpful in a proportion of patients, hence early antibacterial treatment to prevent permanent joint damage remains a challenge (1, 3).

Reports from patients diagnosed with SA caused by uncommon agents has been on the rise (4). *Streptococcus agalactiae* is one of such organisms. *S. agalactiae*, group B streptococcus, colonizes the genitourinary tract in women of child bearing age as the normal flora, and frequently leads to infection in pregnant and puerperal women (5). Under certain environmental changes it harbors virulence factors and may lead to invasive infections (6). Underlying patient conditions exacerbated the presentation of such infections; diabetes mellitus remains a major risk factor (7).

A review introduced 147 cases of *S. agalactaie* arthritis until 2008; most of them documented after the 1980’s (8). More recent reports confirm a similar trend (9, 10). In the current study, we report a rare case of bilateral knee arthritis caused by *S. agalactiae*.

## CASE REPORT

Our patient was a 59 year woman with right knee arthropathy; she could barely only move on her knees 5-months prior to admission. On 12 March 2016, she developed right knee pain and was immobilized in a week. Non-purulent synovial fluid was aspirated from the joint (no laboratory evaluation); she was discharged with painful knee and diagnosis of osteoarthritis. Shortly, she developed left knee pain and swelling. On day 11, a second synovial fluid aspiration revealed purulent discharge with Gram-positive cocci (152,000 WBCs and 70% PMNs) (Table 1).

**Table 1.** Synovial fluid analysis from right knee aspiration on 2^nd^ admission.

**Synovial fluid analysis**	**Result**	**Unit**	**Reference range**
Color and transparency	Milky & Turbid	Descriptive	Colorless & transparent
Mucin clot test	Few	Descriptive	Fair to weak clot indicates inflammatory process
Total leukocyte count	152000	/uL	150–200
RBC	450000	/uL	0
Neutrophil	70	%	Inflammatory>50%
			Septic>90%
Monocyte and macrophage	27	%	Around 65% in normal cell count
Lymphocyte	3	%	Around 15%
Synovial glucose	15	mg/dL	Normal serum/synovial difference<10
Synovial protein	2.8	gr/dL	1–3

She had a history of type 2 diabetes, hypertension, and hyperlipidemia. She had had right-eye evacuation surgery, left-eye cataract surgery, and an amputated 5^th^ finger. She was under treatment with insulin, losartan, captopril, diltiazem, aspirin, atorvastatin, and imipramine. Vital signs were as follows; blood pressure: 140/90 mmHg, heart rate: 100/minute, respiratory rate: 32/minute, and body temperature: 37.4°C. Swelling and pain on active and passive movements were present in both knees.

The patient had leukocytosis, normochromic normocytic anemia; ESR was reported to be 120, quantitative CRP was measured to be 121; blood culture, and all viral markers were negative. After knee arthrotomy on day 6 of third admission, cloudy, purulent discharge was sent for smear and culture analysis (WBC: 92,000; PMN: 90%) with Gram-positive cocci in the smear and a negative culture. Diagnostic tap of the left knee showed 14,480 WBCs (PMN: 95%). Both smear and culture studies were negative. Left knee arthrotomy showed purulent discharge. We found two positive blood and one synovial fluid cultures for *S. agalactiae* in previous hospital records (Table 2).

**Table 2.** Synovial fluid bacteriology analysis from right knee aspiration on 2^nd^ admission.

**Test**	**Result**
Gram smear	Gram positive cocci
Culture	Streptococcus group B (*agalactiae*)
Sensitive to	Ampicilin-Vancomycin-Linezolid-Clindamycin-Erythromycin-Meropenem-Cefepir-Penicilin-Ceftazidime-Levofloxacin-Chloramphenicol-Ofloxacin-Ciprofloxacin
Intermediate to	-
Resistant to	Co-trimoxazole-Tetracyclin

Echocardiography, urine culture, chest x-ray and abdominopelvic ultrasound showed no pathologies.

Vancomycin (1 g daily) was prescribed based on the initial smear and renal adjustment (GFR=32), then replaced with ampicillin (2 g three times daily) for 4 weeks (after the culture results). In follow up, leukocystosis, ESR and CRP levels were declined. Since she was not ambulant at baseline, full recovery was not expected and potential functional sequels of SA were not possible to be assessed.

## DISCUSSION

We report a case of bilateral knee arthritis caused by *S. agalactiae* in a 59-year-old diabetic woman. *S. agalactiae* is a rare cause of infection in non-pregant women with increasing rates in recent years (8). The organism is generally known as normal flora of the genitourinary tract of women of reproductive and at times non-reproductive age (5). Under certain circumstances the organism expresses virulence factors such as the polysaccharide capsule which attacks the innate immune system of the host and leads to serious infections; diabetes mellitus is one such predisposing disorder (4–7). SA being one of these infections is of prominent importance because of permanent and destructive complications (3).

Streptococcal SA has been associated with a certain pattern of joint involvement. They commonly cause oligo or polyarthritis in women, and are associated with older age (12). Our patient was a middle-age woman with oligoarthritis. Positive blood culture is more observed with streptococcal infections; the same observation was reported in our patient considering several negative synovial fluid tap cultures and two positive blood cultures in the second admission (12, 13). Streptococci commonly affect the knee (12, 13), however, GBS has tropism for axial joints; which is not observed in our case (9, 10, 14). A previous study introduces a patient with GBS knee arthritis, however, the joint was prosthetic and prior surgery seemed to be the predisposing factor (4); our patient had knee arthropathy but no history of surgery. However, based on the existing evidence diabetes mellitus may have been the major predisposing condition. Insidious course and milder symptoms are reported with GBS arthritis (12). Our patient had a fluctuating course, possibly because of incomplete treatment before definitive diagnosis. Auto-immune and lymphoproliferative disorders, hepatitis C, and malignancy have been documented as precipitating factors (11, 14). Knee arthritis has been reported as a presentation of tonsillar carcinoma; with similar age and course compared to our study (11). However, we found no specific indicators of malignancy. Dental procedures or other evident points of entry have been suggested, though not seen in our patient (8). Our patient was immunocompetent; diabetes mellitus and osteoarthritis of the knees seemed to be the main susceptibility factors.

The study highlights the importance of considering rare causes of septic arthritis in elderly women who suffer from major comorbid conditions such as diabetes mellitus when presenting to the emergency department.
